# Association between stress hyperglycemia ratio and all-cause mortality among ICU patients with sepsis: a systematic review and meta-analysis

**DOI:** 10.3389/fmed.2025.1741993

**Published:** 2026-01-05

**Authors:** Haohao Xie, Tian Hao, Ruirui Qi, Lin Zhang, Yuyan An, Donghui Jia, Hengyang Wang, Wenxiu Niu, Xiaomeng Han, Yuhan Sha, Li Yang, Zhigang Zhang

**Affiliations:** 1 School of Nursing, Lanzhou University, Lanzhou, Gansu, China; 2 Department of Critical Care Medicine, The First Hospital of Lanzhou University, Lanzhou, Gansu, China

**Keywords:** all-cause mortality, intensive care unit, meta-analysis, prognosis, sepsis, stress hyperglycemia ratio (SHR), systematic review

## Abstract

**Background:**

The stress hyperglycemia ratio (SHR) is defined as the admission blood glucose level divided by the estimated average glucose derived from glycated hemoglobin (HbA1c). Previous studies have demonstrated that higher SHR levels are associated with increased all-cause mortality among intensive care unit (ICU) patients. However, the relationship between SHR and mortality risk specifically in patients with sepsis remains controversial.

**Objectives:**

This study aimed to systematically evaluate, through a systematic review and meta-analysis, the association between SHR and all-cause mortality among adult ICU patients with sepsis.

**Methods:**

A comprehensive search was performed in PubMed, Web of Science, Embase, and the Cochrane Library databases. The methodological quality of included studies was assessed using the Newcastle–Ottawa Scale (NOS). A random-effects model was employed to pool relative risks (RR) with corresponding 95% confidence intervals (CIs). All statistical analyses were conducted using Stata version 18.0.

**Results:**

A total of 11 retrospective cohort studies comprising 37,790 participants were included. Pooled analyses showed that higher SHR levels were significantly associated with increased risks of in-hospital mortality (RR = 2.11, 95% CI: 1.79–2.50; *I*^2^ = 36.3%), short-term mortality (RR = 1.56, 95% CI: 1.38–1.77; *I*^2^ = 0%), and long-term mortality (RR = 1.52, 95% CI: 1.40–1.65; *I*^2^ = 10.3%). Subgroup analyses based on follow-up duration (60 days, 90 days, and 1 year) revealed no statistically significant differences in effect size (*p* = 0.511), suggesting that follow-up duration was not a major source of heterogeneity. Meta-regression analysis indicated that studies with a higher proportion of diabetic patients showed a stronger association between SHR and in-hospital mortality (*p* = 0.026). The overall methodological quality of the included studies was high.

**Conclusion:**

This systematic review and meta-analysis demonstrated that elevated SHR is an independent predictor of in-hospital, short-term, and long-term all-cause mortality among ICU patients with sepsis. SHR, as a simple and valuable prognostic biomarker, may aid in early risk stratification of patients with sepsis.

**Systematic review registration:**

https://www.crd.york.ac.uk/PROSPERO/view/CRD420251139874, Identifier CRD420251139874.

## Highlights

(i) What is currently known about this topic?
 o Stress hyperglycemia is common in critically ill patients and linked to poor outcomes.
 o SHR adjusts acute glucose for chronic glycemia, improving prognostic accuracy.
 o Evidence on SHR and mortality in sepsis remains limited and inconsistent.
(ii) What is the key research question?
 o Is elevated SHR independently associated with increased mortality among ICU patients with sepsis?
(iii) What is new?
 o First meta-analysis confirming higher SHR predicts in-hospital, short- and long-term mortality.
 o The association is stronger in studies with higher proportions of diabetic patients.
 o SHR provides consistent prognostic value regardless of follow-up duration.
(iv) How might this study influence clinical practice?
 o SHR may serve as a simple, low-cost biomarker for early risk stratification in sepsis.

## 1 Introduction

Sepsis is a life-threatening syndrome of organ dysfunction resulting from a dysregulated host response to infection (1). It remains one of the most common and fatal critical illnesses in the intensive care unit (ICU). Despite continuous advancements in early recognition, infection control, and organ support strategies, sepsis still poses a major global health challenge, with an estimated 49 million cases and more than 11 million related deaths annually (2). Its mortality rate, exceeding 25%, imposes a substantial burden on public health systems worldwide and underscores the importance of early identification and risk stratification (3). Therefore, developing novel, simple, and noninvasive biomarkers to facilitate risk stratification and prognostic assessment in patients with sepsis holds significant clinical relevance.

With increasing understanding of the pathophysiology of sepsis, glucose metabolism dysregulation has been recognized as an important determinant of patient outcomes (4). Sepsis triggers an intense stress response through immune dysregulation, systemic inflammation, and multiorgan dysfunction, leading to stress-induced hyperglycemia (SIH) (5, 6). SIH is a common metabolic reaction in acute critical illness (such as sepsis), mediated by the release of stress hormones including epinephrine and cortisol, accompanied by insulin resistance and enhanced hepatic gluconeogenesis (7, 8). While moderate hyperglycemia can provide energy to immune cells and vital organs, excessive or prolonged hyperglycemia promotes oxidative stress, endothelial dysfunction, and immunosuppression, ultimately exacerbating organ injury and increasing the risk of death (9, 10).

In recent years, the stress hyperglycemia ratio (SHR) has been proposed as a novel biomarker to quantify acute stress-induced hyperglycemia. SHR is defined as the ratio of admission blood glucose to estimated average glucose derived from glycated hemoglobin (HbA1c), thereby correcting for chronic hyperglycemia and more accurately reflecting the intensity of acute metabolic stress (11). Its calculation relies solely on two routine laboratory parameters—blood glucose and HbA1c—making it simple, cost-effective, and highly reproducible. Multiple studies have demonstrated the prognostic significance of SHR across various critical illnesses. A recent Meta-analysis reported that elevated SHR was significantly associated with both short-term and long-term all-cause mortality in patients with cardiovascular diseases (4). Moreover, in patients with sepsis-associated acute kidney injury, a U-shaped relationship was observed between SHR and 30-day mortality (12). Previous findings have indicated that SHR shows superior sensitivity and specificity for predicting outcomes in critically ill patients compared with admission glucose or HbA1c alone, suggesting its potential clinical utility for risk stratification and glycemic management in sepsis (13).

Although several studies have investigated the prognostic value of SHR in various critical conditions, evidence specific to ICU patients with sepsis remains limited and inconsistent. To date, no comprehensive synthesis has clarified the relationship between SHR and mortality risk in this population. Therefore, this study aimed to perform a systematic review and meta-analysis to evaluate the association between SHR and all-cause mortality among ICU patients with sepsis, providing evidence-based insights to support clinical decision-making in risk stratification and glucose management.

## 2 Methods

This systematic review and meta-analysis was conducted in accordance with the Preferred Reporting Items for Systematic Reviews and Meta-Analyses (PRISMA) 2020 statement (45). The study protocol was prospectively registered in the International Prospective Register of Systematic Reviews (PROSPERO; registration number: CRD420251139874).

### 2.1 Data sources and search strategy

A comprehensive literature search was performed in PubMed, Embase, Web of Science, and the Cochrane Library, covering all publications from database inception to September 1, 2025. The primary search terms included: “Sepsis” AND “Stress Hyperglycemia Ratio.” Detailed search strategies for each database are provided in Supplementary material 1.

### 2.2 Eligibility criteria

Studies were included if they met the following criteria:

(1) Participants were adult patients (≥18 years) diagnosed with sepsis or septic shock and admitted to the intensive care unit (ICU).
(2) SHR was used as an exposure variable, analyzed either as a categorical variable (e.g., high vs. low SHR groups) or as a continuous variable.
(3) Reported effect estimates for the association between SHR and all-cause mortality, expressed as relative risk (RR), odds ratio (OR), or hazard ratio (HR) with corresponding 95% confidence intervals (CIs).
(4) Adopted an observational design, including cross-sectional, case–control, or cohort studies.

Exclusion criteria were:

(1) Reviews, editorials, letters, conference abstracts, case reports, duplicate publications, or studies with incomplete data.
(2) Non-English articles or animal studies.

### 2.3 Literature screening and data extraction

Two reviewers (HX and TH) independently screened titles and abstracts to exclude duplicates and irrelevant studies. Full-text articles were then assessed for eligibility. Disagreements were resolved through discussion or consultation with a third reviewer (RQ). Data were independently extracted by two reviewers using a standardized form, including: first author’s name and publication year; country and study design; sample size and population characteristics (e.g., mean age and female ratio); diagnostic criteria for sepsis; number of deaths and effect estimates; outcome measures and adjustment for confounding factors.

### 2.4 Risk of bias assessment

The methodological quality of included studies was independently assessed by two reviewers (HX and TH), with disagreements resolved through discussion with a third author (RQ). The Newcastle–Ottawa Scale (NOS) was used to evaluate study quality across three domains: selection of participants, comparability of study groups, and ascertainment of exposure or outcomes. Scores of 0–4, 5–6, and 7–9 were categorized as low, moderate, and high quality, respectively.

### 2.5 Statistical analysis

All statistical analyses were conducted using Stata version 18.0 (StataCorp., College Station, TX, United States). The primary effect measure was the relative risk (RR) with corresponding 95% confidence intervals (CIs). Pooled estimates were calculated using a random-effects model to account for potential between-study heterogeneity. A comprehensive meta-analytic approach was employed to estimate pooled effect sizes. To explore the dose–response relationship between SHR levels and mortality risk, the Greenland and Longnecker (14) method and the approach proposed by Orsini et al. (15) were applied. For studies that reported at least three SHR categories, the median value of each category was used as the exposure level. When only ranges were provided, the midpoint of the upper and lower boundaries was used as the estimated median. Publication bias was assessed visually using funnel plots and statistically using Egger’s regression test (performed when ≥10 studies were included). Sensitivity analyses were conducted using the leave-one-out method to evaluate the stability of pooled results. A two-tailed *p*-value < 0.05 was considered statistically significant.

## 3 Results

### 3.1 Study selection and inclusion

**Figure 1:**
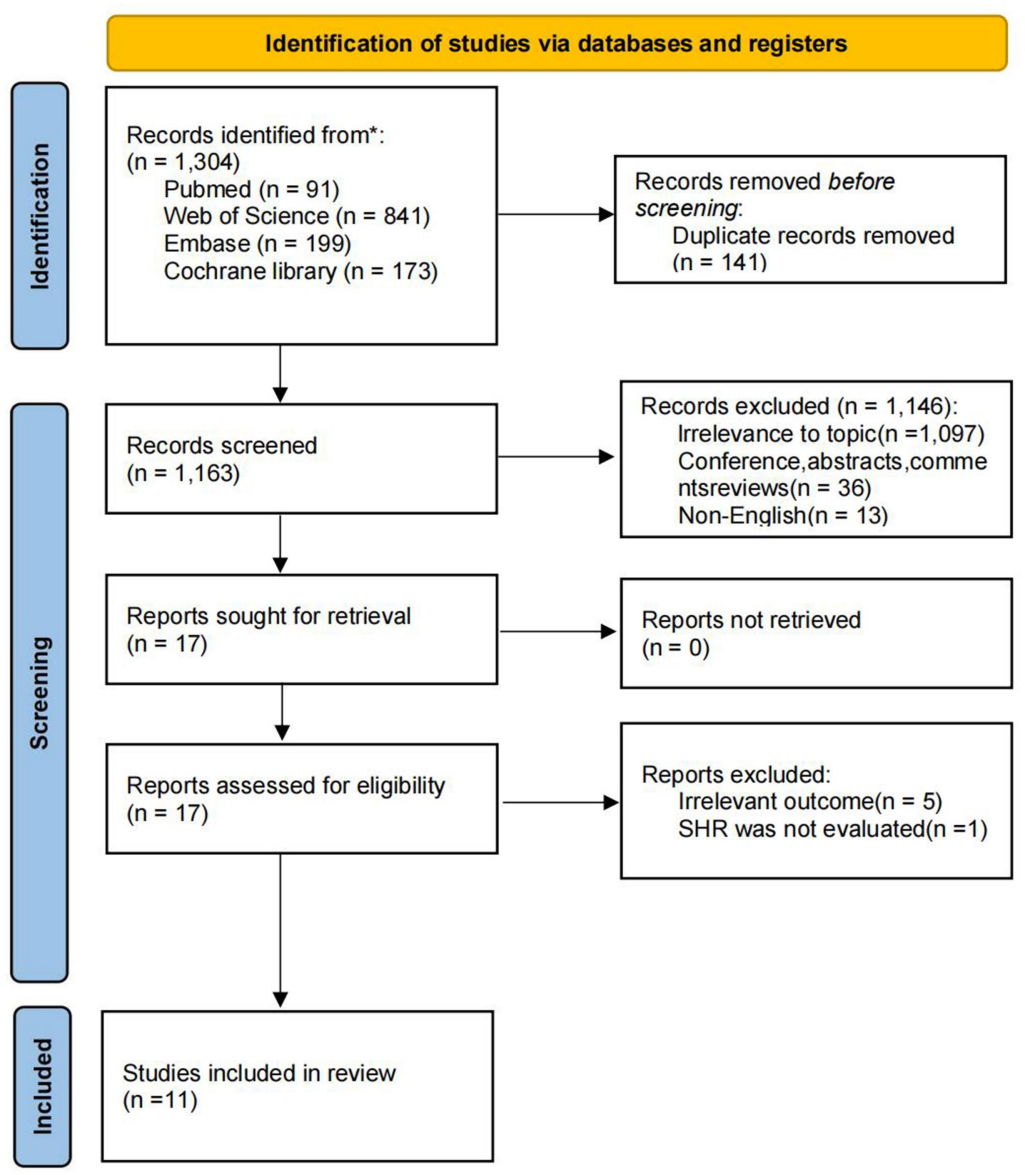
Flow diagram of studies identified for the systematic review and meta-analysis.

A total of 1,304 records were identified through systematic database searching. After removing 141 duplicates and excluding 1,163 irrelevant articles during title and abstract screening, 17 full-text studies were assessed for eligibility. Ultimately, 11 studies met the inclusion criteria and were included in the meta-analysis. The detailed screening and selection process is illustrated in Figure 1.

### 3.2 Characteristics of the included studies

The baseline characteristics of the included studies are summarized in Table 1. A total of 37,790 participants were enrolled across the 11 included retrospective cohort studies, all published between 2024 and 2025. Among these, 7 studies reported data on in-hospital mortality, 7 studies on short-term mortality, and 8 studies on long-term mortality. Most studies (*n* = 10, 90.9%) utilized data from the MIMIC-IV database, while one study was based on a dual-center Chinese cohort. The mean age of participants ranged from 63.24 to 72.0 years, with 40.04% female patients. All studies defined sepsis according to the sepsis-3 criteria and adjusted for multiple potential confounders, including age, sex, comorbidities (e.g., diabetes mellitus, coronary artery disease, hypertension, chronic kidney disease), vital signs, and laboratory parameters.

**Table 1:** Characteristics of included studies.

Study	Cohort-based population	Study design/Database	Patients characteristic	Number of participants	Mean age (years)	Male/Female	Diabetes %	Follow up	Outcomes	Adjustment
Xia et al. (5)	USA	RO/MIMIC-IV	Critically ill sepsis patients	2,407	67	1,433/974	41.9%	90 days	28-day, 60-day & 90-day all-cause mortality	DM, CVD, CRRT, SAPS II, OASIS, CCI
Zhang (46)	USA	RO/MIMIC-IV	Critically ill sepsis patients	7,456	65.37	4,379/3,077	32.3%	1 year	In-hospital &1 year all-cause mortality	SOFA, race, hypertension, history of myocardial infarction, CHD, heart failure, CVD, DM, chronic lung disease, CKD
Zhou et al. (40)	USA	RO/MIMIC-IV	Critically ill SA-AKI patients	1,161	69	665/496	11.4%	1 year	30-day & 1 year all-cause mortality	Age, sex, race, HF, CKD, CVD, DM, cancer, CLD, HR, MBP, RDW, PLT, Hb, AG, HCO₃^−^, BUN, Na, K, PT, ALT, AST, culture, CRRT, insulin, inotropes, vasopressors, steroids, AKI stage, SOFA, SAPS II
Song et al. (7)	USA	RO/MIMIC-IV	Critically ill sepsis and HF patients	869	72	519/350	52.5%	28 days	28-day, In-hospital & ICU all-cause mortality	Age, temperature, oxygen saturation, CVD, dementia, renal disease, mild liver disease, urine output, antibiotic use, SOFA, SAPS II, APACHE II, LODS
Yan et al. (9)	USA	RO/MIMIC-IV	Critically ill sepsis patients	2,312	65	1,396/916	39.2%	28 days	28-day & In-hospital all-cause mortality	Age, weight, sex, HR, RR, SBP, SOFA, steroid use
Ma et al. (44)	China	RO	Critically ill sepsis patients	1,835	71	1,157/678	40.5%	60 days	In-hospital, 30-day & 60-day all-cause mortality	Age, Gender, Scr, C-reactive protein, WBC, Hct, HTN, CVD, CKD, MV
Li et al. (10)	USA	RO/MIMIC-IV	Critically ill sepsis patients	13,199	68.3	8,021/5,178	47.8%	1 year	In-hospital & 1 year all-cause mortality	Age, sex, weight, urine output, HF, DM, HTN, pneumonia, stroke, AKI, CKD, liver disease, cancer, dyslipidemia, anemia, WBC, Scr, BUN, insulin, antibiotics, MV, CRRT
Zhang (47)	USA	RO/MIMIC-IV	Critically ill SA-AKI patients	1,822	68	1,059/763	33.7%	1 year	30-day all-cause mortality	Gender, marital, HTN, DM, care unit, CRRT, vasoactive use, invasive MV, age, BMI, SOFA, HR, MBP, RR, temperature, SpO₂
Zuo (48)	USA	RO/MIMIC-IV	Critically ill sepsis patients	4,276	63.24	2,553/1,723	25.4%	1 year	In-hospital, 90-day & 1 year all-cause mortality	Cardiomyopathy, CHD, AMI, ARF, AKF, HR, RR, SOFA, AST, WBC, Scr, BUN, K, Ca, lactate, Cl^−^, LDL, MV, glucocorticoids
Feng (49)	USA	RO/MIMIC-IV	Critically ill sepsis patients	1,200	68.44	679/521	0	In-hospital	In-hospital & ICU all-cause mortality	Age, sex, weight, WBC, PLT, RBC, RDW, MCV, Hb, HbA1c, glucose, AG, HCO₃^−^, BUN, Cl^−^, Scr, Na, K, Ca, Mg, INR, PT, PTT, ALT, ALP, AST, TBil, SBP, DBP, HR, RR, HTN, HF, AMI, stroke, COPD, renal failure, CLD, malignant cancer, SOFA, APS III, GCS
Wang (50)	USA	RO/MIMIC-IV	Critically ill sepsis patients	1,253	66.84	798/455	0	90 days	28-day & 90-day all-cause mortality	Age, sex, race, BMI, HbA1c, Scr, Hb, GFR, BUN, CVD, PVD, pulmonary disease, HTN, AKI

AG, anion gap; AKI, acute kidney injury; ALP, alkaline phosphatase; ALT, alanine aminotransferase; AMI, acute myocardial infarction; APACHE III, Acute Physiology and Chronic Health Evaluation III; APS III, Acute Physiology Score III; ARF, acute respiratory failure; AST, aspartate aminotransferase; BMI, body mass index; BUN, blood urea nitrogen; Ca, calcium; CCI, Charlson comorbidity index; CHD, coronary artery disease; CKD, chronic kidney disease; Cl^−^, chloride; CLD, chronic liver disease; COPD, chronic obstructive pulmonary disease; CRP, C-reactive protein; CRRT, continuous renal replacement therapy; CVD, cardiovascular disease; DBP, diastolic blood pressure; DM, diabetes mellitus; GCS, Glasgow Coma Scale; GFR, glomerular filtration rate; Hb, hemoglobin; HbA1c, glycated hemoglobin; HCO₃^−^, bicarbonate; Hct, hematocrit; HF, heart failure; HR, heart rate; HTN, hypertension; ICU, intensive care unit; INR, international normalized ratio; K, potassium; LDL, low-density lipoprotein; LODS, logistic organ dysfunction score; MBP, mean blood pressure; MCV, mean corpuscular volume; Mg, magnesium; MIMIC-IV, Medical Information Mart for Intensive Care; MV, mechanical ventilation; Na, sodium; OASIS, Oxford Acute Severity of Illness Score; PLT, platelet count; PT, prothrombin time; PTT, partial thromboplastin time; PVD, peripheral vascular disease; RBC, red blood cell count; RDW, red cell distribution width; RO, retrospective cohort study; RR, respiratory rate; SA-AKI, sepsis-associated acute kidney injury; SAPS II, Simplified Acute Physiology Score II; SBP, systolic blood pressure; Scr, serum creatinine; SOFA, Sequential Organ Failure Assessment; SpO₂, peripheral capillary oxygen saturation; TBil, total bilirubin; Temp, temperature; UO, urine output; WBC, white blood cell count.

### 3.3 Methodological quality of the included studies

According to the Newcastle–Ottawa Scale (NOS), 10 studies achieved a score of 9, and one study scored 8, indicating an overall high methodological quality. Detailed quality assessment results are provided in Supplementary material 2.

### 3.4 Association between SHR and in-hospital mortality

**Figure 2:**
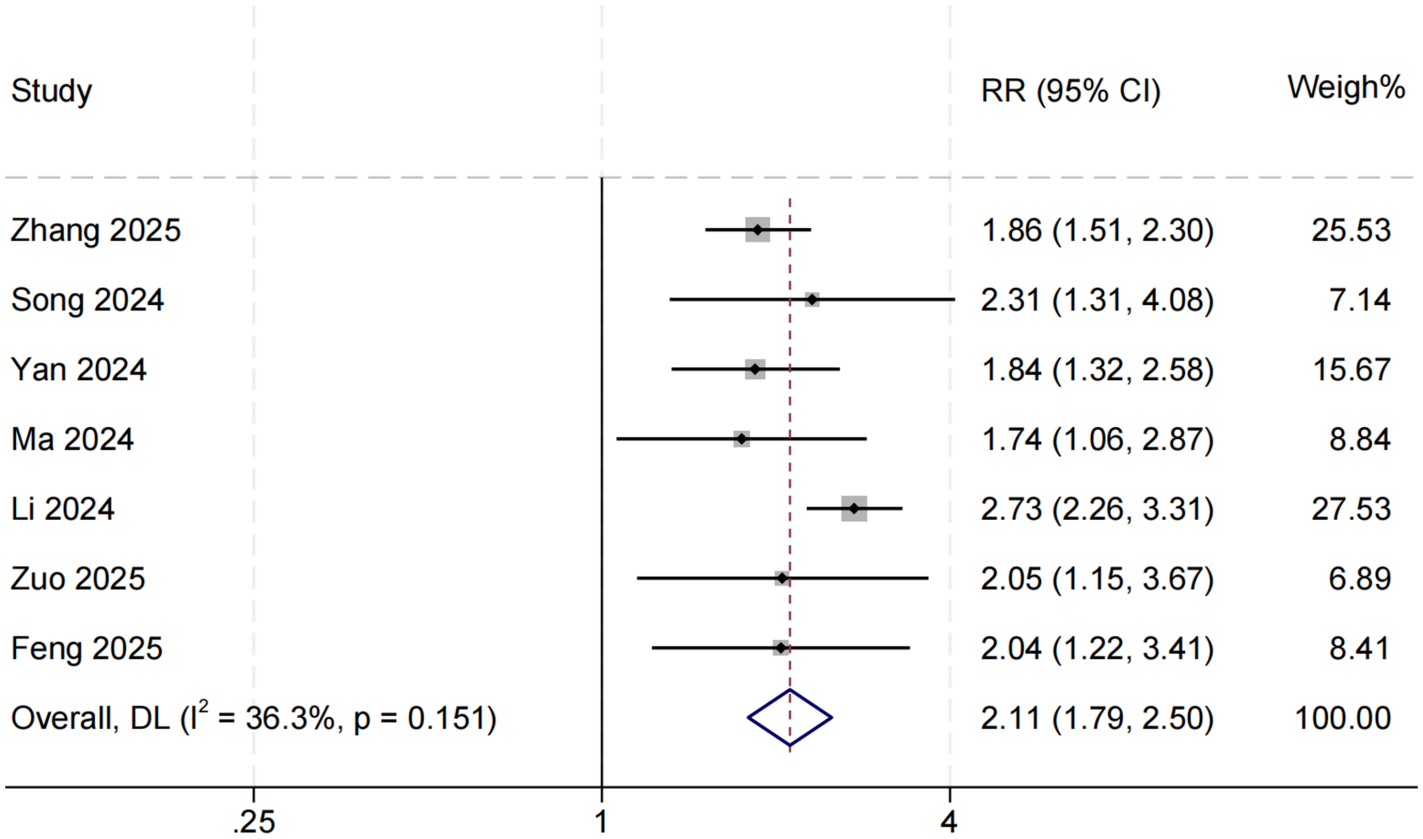
SHR and in-hospital all-cause mortality. Weights are from random-effects model.

Seven studies evaluated the association between SHR and in-hospital all-cause mortality among patients with sepsis in the ICU. The pooled results demonstrated that higher SHR was significantly associated with increased in-hospital mortality (RR = 2.11, 95% CI: 1.79–2.50, *p* < 0.001; *I*^2^ = 36.3%, *p* = 0.151) (Figure 2). To further explore potential sources of heterogeneity, meta-regression analyses were performed based on female proportion, mean age, prevalence of diabetes mellitus, and mean SOFA score. The association between SHR and in-hospital mortality was found to be stronger in studies with a higher proportion of diabetic patients (*p* = 0.026). Interestingly, in studies involving patients with higher SOFA scores—indicating greater illness severity—the strength of this association was attenuated (*p* = 0.068). Moreover, a trend toward a stronger association was observed in studies with a higher proportion of female participants (*p* = 0.070). Detailed meta-regression results are presented in Table 2.

**Table 2:** Meta-regression of SHR with in-hospital mortality.

Parameter	Number of included studies	Effect size	95% CI	*p*-value
In-hospital mortality				
Female (%)	7	20.799	(−1.67, 43.27)	0.070
Age (mean)	7	0.0215	(−0.04, 0.09)	0.504
Diabetes (%)	7	2.504	(0.30, 4.71)	0.026
SOFA score (mean)	7	−0.283	(−0.59, 0.02)	0.068

CI, confidence interval; SOFA, Sequential Organ Failure Assessment.

### 3.5 Association between SHR and short-term mortality

**Figure 3:**
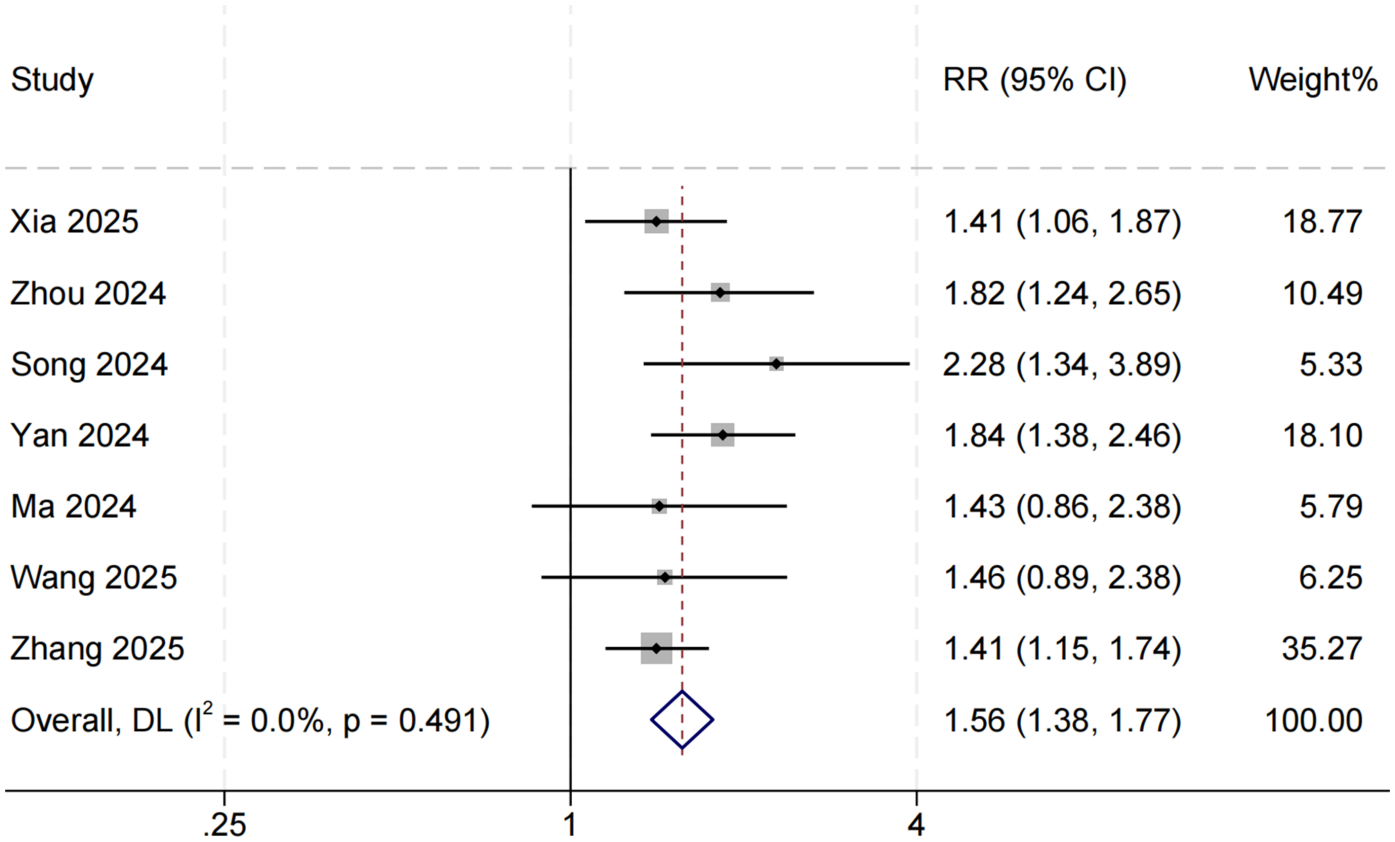
SHR and short-term all-cause mortality. Weights are from random-effects model.

Seven studies assessed the relationship between SHR and short-term all-cause mortality in critically ill patients with sepsis. The pooled results indicated that elevated SHR was significantly associated with an increased risk of short-term mortality (RR = 1.56, 95% CI: 1.38–1.77, *p* < 0.001; *I*^2^ = 0%, *p* = 0.491) (Figure 3).

### 3.6 Association between SHR and long-term mortality

**Figure 4:**
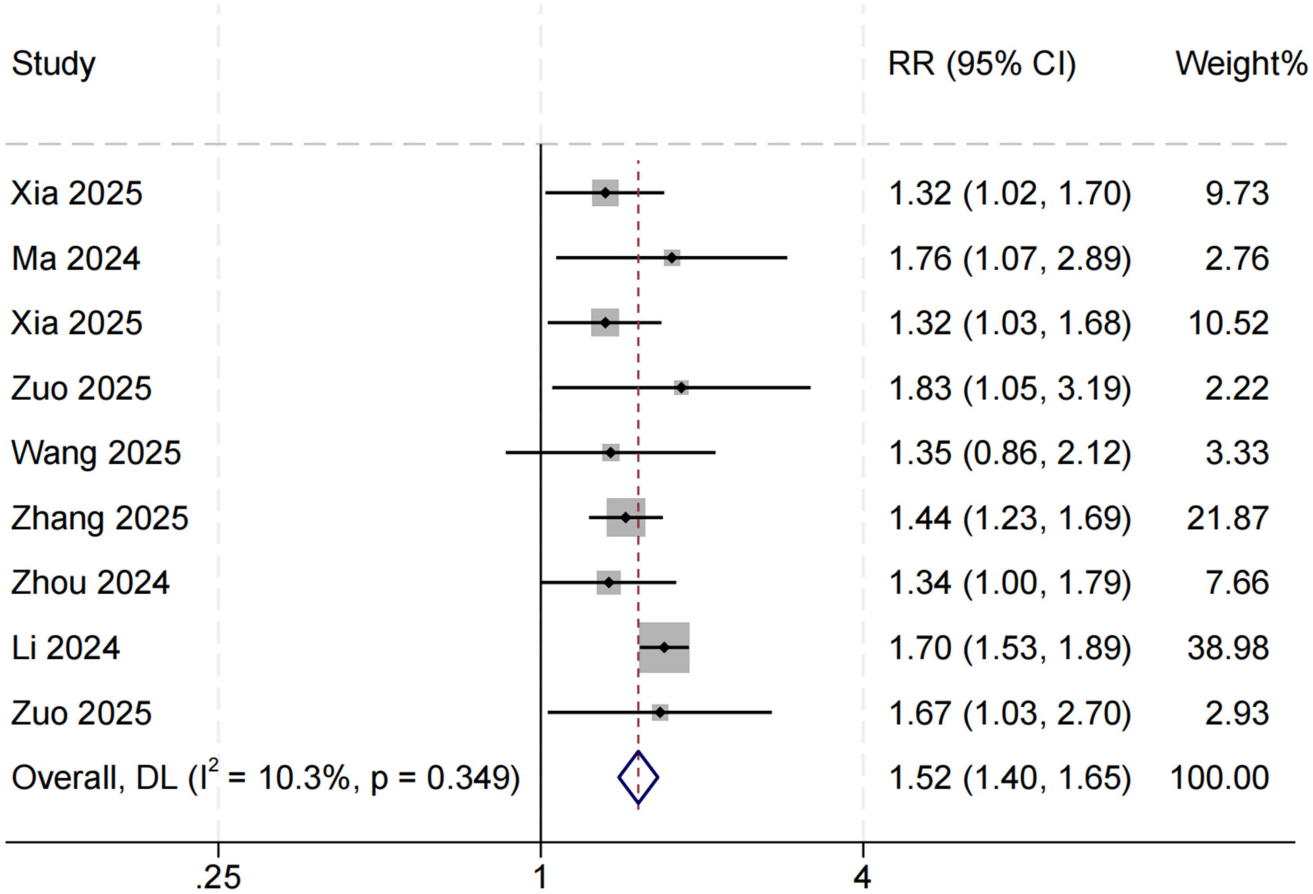
SHR and long-term all-cause mortality. Weights are from random-effects model.

**Figure 5:**
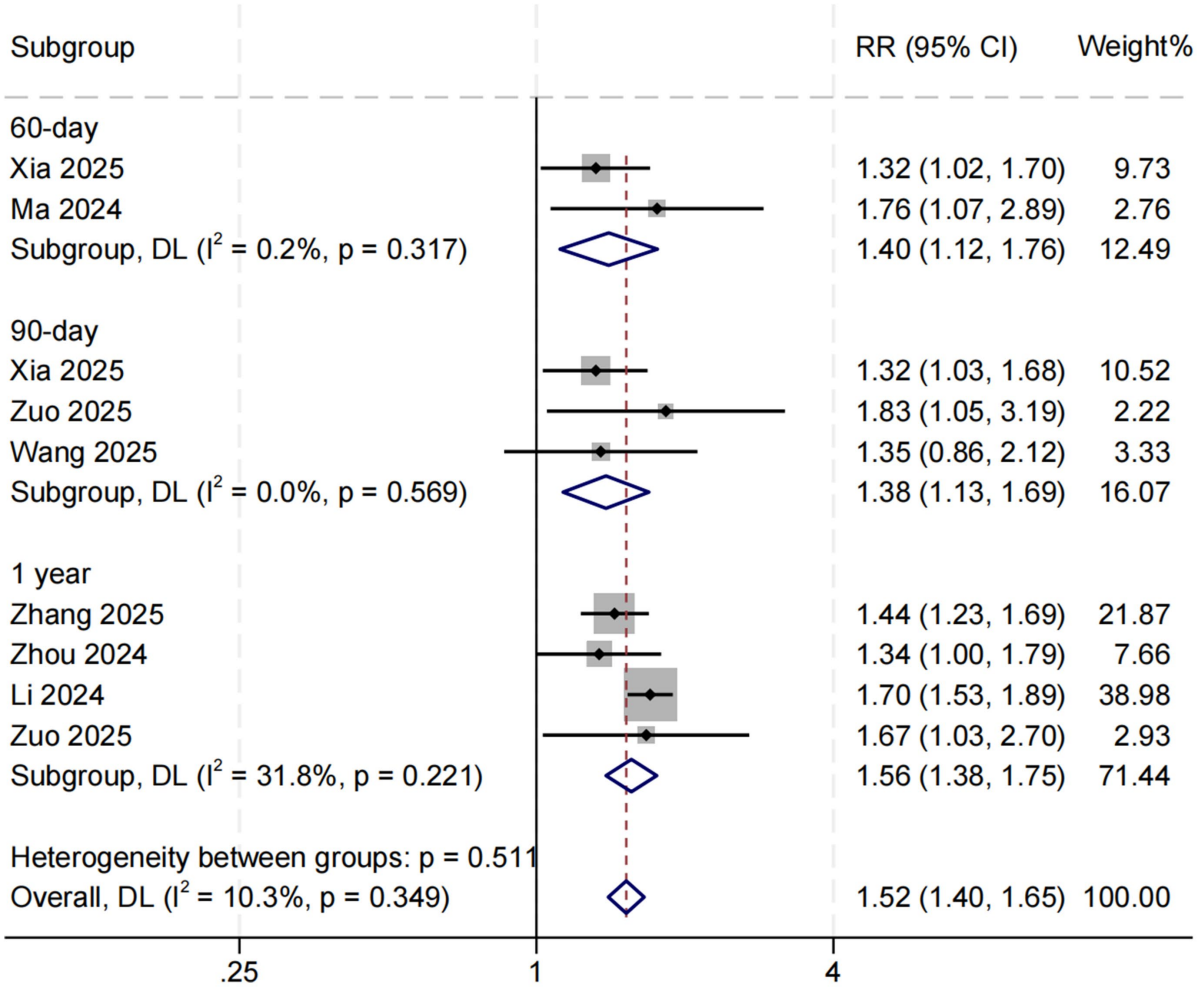
Subgroup analysis for SHR and long-term all-cause mortality. Weights and between-subgroup heterogeneity test are from random-effects model.

Eight studies examined the association between SHR and long-term all-cause mortality among ICU patients with sepsis. The pooled results demonstrated a significant relationship between higher SHR and increased long-term mortality risk (RR = 1.52, 95% CI: 1.40–1.65, *p* < 0.001; *I*^2^ = 10.3%, *p* = 0.349) (Figure 4). Subgroup analyses based on follow-up duration (60 days, 90 days, and 1 year) revealed no significant differences in effect size (*p* = 0.511), suggesting that follow-up duration was not a major source of heterogeneity (Figure 5).

### 3.7 Sensitivity analysis

Sensitivity analyses using the leave-one-out method confirmed the stability and robustness of the pooled results. The associations between SHR and in-hospital (95% CI: 0.64–0.81), short-term (95% CI: 0.58–0.91), and long-term (95% CI: 0.335–0.503) all-cause mortality remained consistent after sequentially omitting individual studies, indicating that no single study substantially influenced the overall findings.

### 3.8 Publication bias

**Figure 6:**
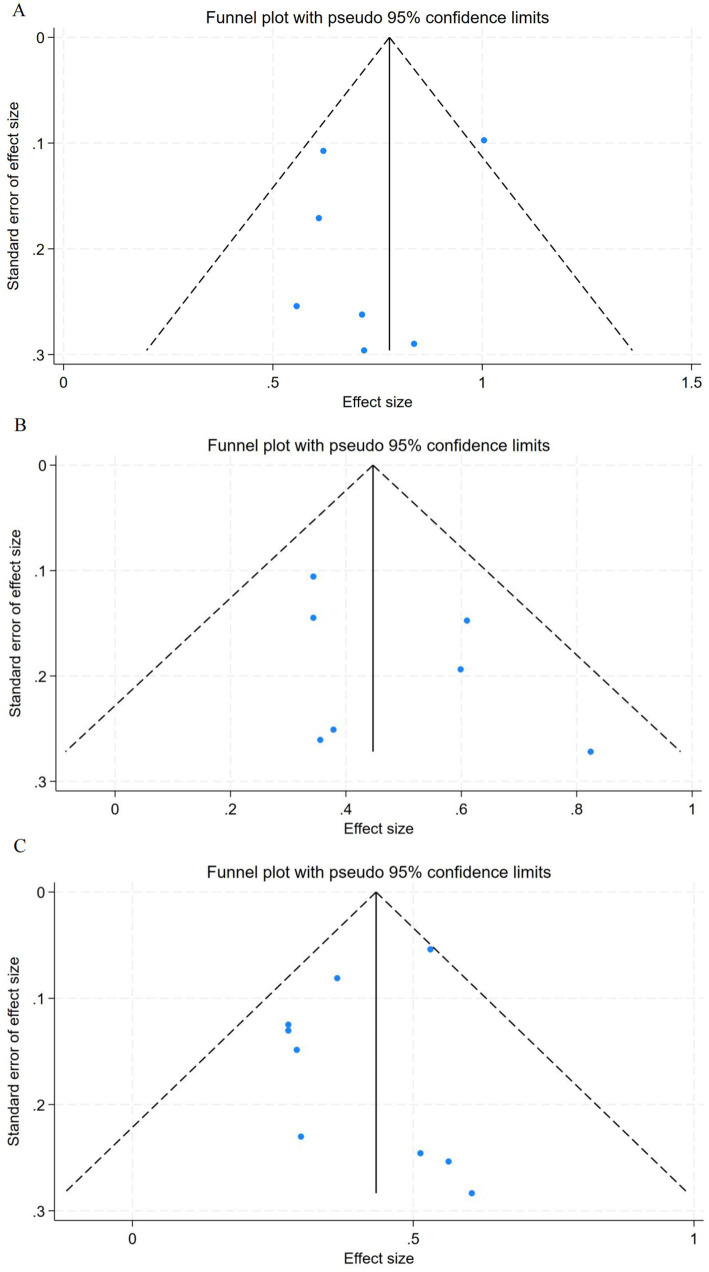
Funnel plots for association between SHR and all-cause mortality. **(A)** In-hospital mortality. **(B)** Short-term mortality. **(C)** Long-term mortality. SHR, stress hyperglycemia ratio.

Visual inspection of funnel plots revealed mild asymmetry among studies assessing the relationship between SHR and in-hospital mortality (Figure 6A). Given the limited number of included studies (*n* = 7), Egger’s regression test was not performed due to insufficient statistical power. For short-term mortality, the funnel plot appeared symmetrical, suggesting no significant publication bias (Figure 6B). In contrast, the funnel plot for long-term mortality (Figure 6C) displayed slight asymmetry, indicating the possible presence of small-study effects or minor publication bias; however, the overall degree of bias was considered low.

## 4 Discussion

This systematic review and meta-analysis comprehensively evaluated the association between the stress hyperglycemia ratio (SHR) and all-cause mortality among patients with sepsis admitted to the intensive care unit (ICU). Eleven studies involving a total of 37,790 participants were included, all demonstrating generally good methodological quality. The pooled results showed that elevated SHR was significantly associated with increased in-hospital, short-term, and long-term mortality among septic patients. Specifically, higher SHR levels were associated with approximately a 111% increase in in-hospital mortality risk (RR = 2.11, 95% CI: 1.79–2.50), as well as 56 and 52% increases in short-term and long-term mortality, respectively.

To further explore sources of heterogeneity, a meta-regression analysis was conducted, revealing that studies with a higher proportion of diabetic patients demonstrated a stronger association between SHR and in-hospital mortality. Overall heterogeneity among included studies was low, and sensitivity analyses confirmed the robustness and consistency of the results, supporting the reliability of our findings. In the meta-regression analysis, the stronger relationship between SHR and in-hospital mortality observed in cohorts with a higher prevalence of diabetes may reflect a heightened metabolic stress response in individuals with preexisting insulin resistance and chronic hyperglycemia. During acute septic insult, these patients may experience greater impairment of glycemic control, leading to marked glucose fluctuations that exacerbate tissue hypoxia, oxidative stress, and microcirculatory dysfunction—ultimately increasing mortality risk (10, 16). Conversely, in studies including patients with higher SOFA scores—indicative of more severe baseline illness—the strength of this association appeared attenuated (*p* = 0.068), though not statistically significant. This trend suggests that in extremely ill patients, multi-organ failure itself may become the dominant determinant of mortality, thereby diminishing the relative contribution of glucose dysregulation (17, 18). Additionally, subgroup analyses revealed no significant difference in effect size across varying follow-up durations (60 days, 90 days, and 1 year), suggesting that the prognostic value of SHR remains consistent over both short- and long-term outcomes. These findings collectively indicate that SHR not only serves as a useful early risk stratification marker but also possesses potential long-term prognostic relevance in patients with sepsis.

Our findings underscore the potential role of SHR as a clinically valuable biomarker for risk stratification and prognostic assessment in ICU patients with sepsis. The observed association between elevated SHR and increased mortality risk is likely mediated through a complex network of metabolic, immune, and microcirculatory interactions (19). Under acute stress conditions, transient hyperglycemia represents an adaptive response, providing additional energy substrates for immune cells and vital organs such as the brain (20). However, when the stress response becomes excessive or sustained, metabolic regulation shifts from compensation to decompensation, characterized by persistent hyperglycemia, aggravated insulin resistance, and dysregulated inflammation (21). In sepsis, the systemic inflammatory response and cytokine storm amplify this maladaptive process, driving the transition from adaptive to pathological metabolic dysregulation (22, 23). Inflammatory mediators such as IL-6 and TNF-α impair insulin signaling through activation of the JNK and NF-κB pathways, leading to decreased peripheral glucose uptake and enhanced hepatic gluconeogenesis (24, 25). This cascade exacerbates insulin resistance and energy imbalance. Such metabolic derangements not only perpetuate hyperglycemia itself but also suppress immune competence—manifested by impaired neutrophil chemotaxis and phagocytosis, and diminished bactericidal activity of macrophages and T cells—thereby increasing susceptibility to secondary infections (26, 27). Moreover, excessive hyperglycemia and oxidative stress contribute to endothelial injury and generation of reactive oxygen species (ROS), promoting the accumulation of advanced glycation end-products (AGEs) and activation of the RAGE signaling pathway (28–30). These changes increase vascular permeability, induce microcirculatory perfusion defects, and exacerbate tissue hypoxia (31). Ultimately, the interplay between metabolic stress, immune dysfunction, and microcirculatory collapse forms a vicious cycle, driving the progression of multiple organ failure and death (32–34). Therefore, an elevated SHR reflects not merely the absolute glucose level but the degree of acute metabolic stress relative to chronic glycemic status (35). As an integrated marker of systemic metabolic and stress load, SHR captures the continuum from metabolic disturbance to immune suppression and microvascular dysfunction, providing a mechanistic rationale for its strong association with adverse outcomes in sepsis.

The findings of this study are broadly consistent with previous evidence from other critically ill populations, while also revealing potential mechanisms that may be unique to sepsis. A large body of research has shown that elevated SHR is closely associated with adverse outcomes across various critical conditions, including cardiovascular and cerebrovascular events. Similarly, previous studies have emphasized that both hyperglycemia and glycemic fluctuations during critical illness are important determinants of outcomes. Mahmoodpoor et al. (36) identified hypoglycemia as a key predictor of mortality among ICU patients receiving insulin therapy, underscoring the complex relationship between glucose management and survival in critical care. In addition, Safari et al. (37) demonstrated that the APACHE II score could effectively predict diabetic ketoacidosis in hyperglycemic emergencies, highlighting the prognostic relevance of metabolic and stress-related parameters in acute illness. For example, a meta-analysis demonstrated that higher SHR levels were significantly linked to increased short- and long-term mortality in patients with acute myocardial infarction, irrespective of diabetes status (4). Similarly, another cohort study identified admission hyperglycemia as an independent predictor of both in-hospital and one-year all-cause mortality among ICU patients (38). Furthermore, a meta-analysis by Chen et al. (39) suggested a nonlinear dose–response relationship between SHR and major adverse cardiovascular events in critically ill patients, indicating a possible threshold effect rather than a simple linear increase in risk. Other analyses have also recognized SHR as an independent predictor of mortality in critical illness (40). However, in contrast to purely cardiovascular or cerebrovascular conditions, sepsis is characterized by concurrent inflammatory and metabolic dysregulation, which may modify the relationship between SHR and clinical outcomes (41). Inflammatory cytokines profoundly disrupt insulin signaling and exacerbate metabolic dysfunction, leading to a more complex and dynamic pattern of stress hyperglycemia in sepsis compared with other critical illnesses (42). A large MIMIC-based cohort study reported a U-shaped association between SHR and both 28-day and in-hospital mortality among patients with severe sepsis, with particularly high SHR values associated with increased adverse outcomes (43). In contrast, a multicenter Chinese cohort found that sepsis mortality increased markedly when SHR exceeded 1.06, but no U-shaped pattern was observed, suggesting that population characteristics or disease stage may influence the SHR–outcome relationship (44). Although previous evidence has generally supported the prognostic value of SHR in diverse critical conditions, its specific role in sepsis—a disease combining profound inflammation and metabolic stress—has not been systematically verified.

Against this background, the present meta-analysis provides the first comprehensive synthesis confirming that elevated SHR is significantly associated with higher in-hospital, short-term, and long-term all-cause mortality among ICU patients with sepsis. These findings provide systematic and evidence-based support for the application of SHR as a prognostic biomarker in infection-related critical illness, thereby filling an important gap in the existing literature. By integrating available studies, our analysis substantiates SHR as a robust and practical indicator of mortality risk in septic patients and extends its prognostic relevance beyond cardiovascular disease populations. This study also provides a novel perspective on metabolic stress assessment in sepsis. Future research should focus on elucidating the dynamic changes of SHR over time and their relationship with clinical outcomes, as well as determining its predictive value across different stages of sepsis and in subpopulations with or without diabetes. Although current clinical workflows seldom include SHR calculation, the index has potential to become a simple and practical bedside biomarker once integrated into hospital information systems. Moreover, prospective studies are warranted to evaluate whether individualized glycemic management strategies guided by SHR can improve patient outcomes. Overall, SHR—owing to its simplicity, low cost, and reproducibility—shows strong potential for clinical implementation as a valuable tool for risk stratification and prognostic evaluation in ICU patients with sepsis.

### 4.1 Limitations

This study has several limitations. First, all included studies were retrospective cohort designs. Although sensitivity analyses demonstrated the robustness of the pooled results, such designs cannot fully eliminate residual confounding, and therefore causality between SHR and mortality cannot be established. Second, the majority of included data were derived from the MIMIC-IV database, which may limit the generalizability of our findings to other regions, healthcare systems, or patient populations. Third, despite general consistency in the calculation of SHR, cutoff values and categorization methods varied among studies, potentially introducing heterogeneity. In addition, the number of included studies was relatively limited, which may reduce the statistical power of the meta-analysis and affect the accuracy of publication bias assessments. Consequently, our conclusions should be interpreted with caution and further validated through large-scale, multicenter, prospective studies with standardized SHR definitions and consistent outcome measures.

## 5 Conclusion

This systematic review and meta-analysis demonstrated that an elevated stress hyperglycemia ratio (SHR) is significantly associated with increased in-hospital, short-term, and long-term all-cause mortality among intensive care unit (ICU) patients with sepsis. SHR serves as an independent predictor of mortality in this population and may provide a valuable, easily obtainable biomarker for early risk stratification. Future prospective and multicenter studies are warranted to further validate its prognostic utility and to explore whether SHR-guided metabolic management can improve clinical outcomes in sepsis.

## Data Availability

The raw data supporting the conclusions of this article will be made available by the authors, without undue reservation.
