# Supplementary material for: Association between stress hyperglycemia ratio and all-cause mortality among ICU patients with sepsis: a systematic review and meta-analysis
Source: Front Med (Lausanne). 2026 Jan 5;12:1741993. doi: 10.3389/fmed.2025.1741993 (PMC12825456; doi:10.3389/fmed.2025.1741993)
Supplement: Supplementary file 1 [file Table_1.DOCX]

**Association Between Stress Hyperglycemia Ratio and All-Cause Mortality Among ICU Patients With Sepsis: A Systematic Review and Meta-Analysis**

**Haohao Xie et al.**

| **Supplementary material 1:Search strategy (using PubMed as an example); Up to 1/9/2025** | |
| --- | --- |
| **PubMed** | ((((((((((((((stress hyperglycemia[Title/Abstract]) OR (stress hyperglycaemia[Title/Abstract])) OR (stress-induced hyperglycemia[Title/Abstract])) OR (stress induced hyperglycemia[Title/Abstract])) OR (stress-induced hyperglycaemia[Title/Abstract])) OR (stress induced hyperglycaemia[Title/Abstract])) OR (admission hyperglycemia[Title/Abstract])) OR (admission hyperglycaemia[Title/Abstract])) OR (admission glucose[Title/Abstract])) OR (stress hyperglycemia ratio[Title/Abstract])) OR (stress hyperglycaemia ratio[Title/Abstract])) OR (glycemic ratio[Title/Abstract])) OR (stress-hyperglycaemia ratio[Title/Abstract])) OR (glycemic gap[Title/Abstract])) AND ((("Sepsis"[Mesh]) OR "Shock, Septic"[Mesh]) OR (((((((((((((Sepsis[Title/Abstract]) OR (Septic shock[Title/Abstract])) OR (Pyohemia[Title/Abstract])) OR (Pyemia[Title/Abstract])) OR (Pyohemias[Title/Abstract])) OR (Pyaemia[Title/Abstract])) OR (Severe Sepsis[Title/Abstract])) OR (Bloodstream Infections[Title/Abstract])) OR (Bloodstream Infection[Title/Abstract])) OR (Blood Poisonings[Title/Abstract])) OR (Blood Poisoning[Title/Abstract])) OR (Septicemias[Title/Abstract])) OR (Septicemia[Title/Abstract]))). |
