# Supplementary material for: Association between stress hyperglycemia ratio and all-cause mortality among ICU patients with sepsis: a systematic review and meta-analysis
Source: Front Med (Lausanne). 2026 Jan 5;12:1741993. doi: 10.3389/fmed.2025.1741993 (PMC12825456; doi:10.3389/fmed.2025.1741993)
Supplement: Supplementary file 2 [file Table_2.DOCX]

Supplementary Materials 2. Results of quality assessment using the Newcastle-Ottawa Scale (NOS).

| Frist author (year) | Selection | Comparability | Exposure | Quality scores |
| --- | --- | --- | --- | --- |
| Xia 2025 | ★★★★ | ★★ | ★★★ | 9/9 |
| Zhang 2025 | ★★★★ | ★★ | ★★★ | 9/9 |
| Zhou 2024 | ★★★★ | ★★ | ★★★ | 9/9 |
| Song 2024 | ★★★★ | ★★ | ★★★ | 9/9 |
| Yan 2024 | ★★★★ | ★★ | ★★★ | 9/9 |
| Ma 2024 | ★★★★ | ★★ | ★★★ | 9/9 |
| Li 2024 | ★★★★ | ★★ | ★★★ | 9/9 |
| Zhang 2025 | ★★★★ | ★★ | ★★★ | 9/9 |
| Zuo 2025 | ★★★★ | ★★ | ★★★ | 9/9 |
| Feng 2025 | ★★★★ | ★★ | ★★★ | 9/9 |
| Wang 2025 | ★★★★ | ★☆ | ★★★ | 8/9 |

***Selection:*** Q1: Representativeness of the Exposed Cohort; Q2: Selection of the Non-Exposed Cohort; Q3: Ascertainment of Exposure; Q4: Demonstration That Outcome of Interest Was Not Present at Start of Study.

***Comparability:*** Q5: Comparability of cohorts on the basis of the design or analysis controlled for confounders.

***Exposure:*** Q6: Assessment of Outcome; Q7: Was Follow-Up Long Enough for Outcomes to Occur; Q8: Adequacy of Follow-Up of Cohorts.

***A score of 0-3 indicates low quality, 4-6 indicates medium quality, and 7-9 indicates high quality.***
