# Supplementary material for: Association between stress hyperglycemia ratio and all-cause mortality among ICU patients with sepsis: a systematic review and meta-analysis
Source: Front Med (Lausanne). 2026 Jan 5;12:1741993. doi: 10.3389/fmed.2025.1741993 (PMC12825456; doi:10.3389/fmed.2025.1741993)
Supplement: Supplementary file 3 [file Table_3.DOCX]

**Supplementary material 3. Included studies and references**

| **Frist author**  **(year)** | **Title** | **Reference** |
| --- | --- | --- |
| Xia 2025 | Assessment of stress hyperglycemia ratio to predict mortality in critically ill patients with sepsis: a retrospective cohort study from the MIMIC-IV database | Xia, D., Luo, X., Zhu, Y., Zhu, J., & Xie, Y. (2025). Assessment of stress hyperglycemia ratio to predict mortality in critically ill patients with sepsis: a retrospective cohort study from the MIMIC-IV database. Front Endocrinol (Lausanne), 16, 1496696. https://doi.org/10.3389/fendo.2025.1496696 |
| Zhang 2025 | Association between stress hyperglycemia ratio and all-cause mortality in critically ill patients with sepsis: results from the MIMIC-IV database | Zhang, S., Shen, H., Wang, Y., Ning, M., Zhou, J., Liang, X., Chang, Y., Gao, W., & Li, T. (2025). Association between stress hyperglycemia ratio and all-cause mortality in critically ill patients with sepsis: results from the MIMIC-IV database. Eur J Med Res, 30(1), 42. https://doi.org/10.1186/s40001-025-02281-4 |
| Zhou 2024 | The association between stress hyperglycemia ratio and clinical outcomes in patients with sepsis-associated acute kidney injury: a secondary analysis of the MIMIC-IV database | Zhou, Y. J., Zhong, L. P., Zhong, Y. T., & Liao, Y. L. (2024). The association between stress hyperglycemia ratio and clinical outcomes in patients with sepsis-associated acute kidney injury: a secondary analysis of the MIMIC-IV database. BMC INFECTIOUS DISEASES, 24(1), Article 1263. https://doi.org/10.1186/s12879-024-10179-5 |
| Song 2024 | Association between stress hyperglycemia ratio and mortality in patients with heart failure complicated by sepsis | Song, L. J., Ying, J. J., Li, M., Weng, C. X., Jia, S. W., Ying, L., & Li, Z. Y. (2024). Association between stress hyperglycemia ratio and mortality in patients with heart failure complicated by sepsis. SCIENTIFIC REPORTS, 14(1), Article 31380. https://doi.org/10.1038/s41598-024-82890-x |
| Yan 2024 | Association between the stress hyperglycemia ratio and 28-day all-cause mortality in critically ill patients with sepsis: a retrospective cohort study and predictive model establishment based on machine learning | Yan, F. J., Chen, X. H., Quan, X. Q., Wang, L. L., Wei, X. Y., & Zhu, J. L. (2024). Association between the stress hyperglycemia ratio and 28-day all-cause mortality in critically ill patients with sepsis: a retrospective cohort study and predictive model establishment based on machine learning. CARDIOVASCULAR DIABETOLOGY, 23(1), Article 163. https://doi.org/10.1186/s12933-024-02265-4 |
| Ma 2024 | Association of Stress Hyperglycemia Ratio and in-Hospital Mortality in Patients with Sepsis: A Two Center Retrospective Cohort Study | Ma, C., Jiang, W., Li, J., Sun, W., Zhang, J., Xu, P., Guo, Y., Ning, N., Li, J., Zhao, B., Mao, E., & Gao, C. (2024). Association of Stress Hyperglycemia Ratio and in-Hospital Mortality in Patients with Sepsis: A Two Center Retrospective Cohort Study. J Inflamm Res, 17, 7939-7950. https://doi.org/10.2147/jir.S476898 |
| Li 2024 | Association of stress hyperglycemia ratio and mortality in patients with sepsis: results from 13,199 patients | Li, L., Zhou, L., Peng, X., Zhang, Z., Zhang, Z., Xiong, Y., Hu, Z., & Yao, Y. (2024). Association of stress hyperglycemia ratio and mortality in patients with sepsis: results from 13,199 patients. INFECTION, 52(5), 1973-1982. https://doi.org/10.1007/s15010-024-02264-3 |
| Zhang 2025 | Association of stress hyperglycemia ratio with mortality in sepsis-associated acute kidney injury: a retrospective analysis of the MIMIC-IV database | Zhang, J., Xia, J., Niu, Z., Zhu, H., & Wang, X. (2025). Association of stress hyperglycemia ratio with mortality in sepsis-associated acute kidney injury: a retrospective analysis of the MIMIC-IV database. Sci Rep, 15(1), 30667. https://doi.org/10.1038/s41598-025-16783-y |
| Zuo 2025 | Joint association of the triglyceride-glucose index and stress hyperglycemia ratio with incidence and mortality risks of new-onset atrial fibrillation during sepsis: a retrospective cohort study | Zuo, Z. H., Zhou, Z. J., Liu, Q., Shi, R. Z., & Wu, T. (2025). Joint association of the triglyceride-glucose index and stress hyperglycemia ratio with incidence and mortality risks of new-onset atrial fibrillation during sepsis: a retrospective cohort study. CARDIOVASCULAR DIABETOLOGY, 24(1), Article 149. https://doi.org/10.1186/s12933-025-02709-5 |
| Feng 2025 | Stress hyperglycemia ratio as a mortality predictor in non-diabetic septic patients: a retrospective cohort analysis | Feng, S., Zou, R., Wang, Y., Huang, Y., Zhou, Q., Huang, Q., & Xu, H. (2025). Stress hyperglycemia ratio as a mortality predictor in non-diabetic septic patients: a retrospective cohort analysis. BMC Infect Dis, 25(1), 752. https://doi.org/10.1186/s12879-025-11151-7 |
| Wang 2025 | Unmasking the Hidden Risk: Stress Hyperglycemia Ratio Strongly Predicts Death in Sepsis-Associated Encephalopathy | Wang, Y., Qin, S., Zheng, J., Zhang, Y., Qin, K., Qin, H., Dai, Q., Liu, X., & Yu, K. (2025). Unmasking the Hidden Risk: Stress Hyperglycemia Ratio Strongly Predicts Death in Sepsis-Associated Encephalopathy. In. |
